# Is race medically relevant? A qualitative study of physicians' attitudes about the role of race in treatment decision-making

**DOI:** 10.1186/1472-6963-11-183

**Published:** 2011-08-05

**Authors:** Shedra Amy Snipes, Sherrill L Sellers, Adebola Odunlami Tafawa, Lisa A Cooper, Julie C Fields, Vence L Bonham

**Affiliations:** 1 Biobehavioral Health, The Pennsylvania State University, 315 Health and Human Development East, University Park, PA 16802, USA; 2 Family Studies and Social Work, Miami University, 501 East High Street, Oxford, Ohio 45056, USA; 3 Social and Behavioral Research Branch, National Human Genome Research Institute, National Institutes of Health, 31 Center Drive, Bethesda, MD 20892, USA; 4 Harvard School of Public Health, 677 Huntington Avenue, Boston, Massachusetts 02115, USA; 5 Johns Hopkins University School of Medicine, 2024 East Monument Street, Suite 2-500 Baltimore, MD 21287, USA

## Abstract

**Background:**

The role of patient race in medical decision-making is heavily debated. While some evidence suggests that patient race can be used by physicians to predict disease risk and determine drug therapy, other studies document bias and stereotyping by physicians based on patient race. It is critical, then, to explore physicians' attitudes regarding the medical relevance of patient race.

**Methods:**

We conducted a qualitative study in the United States using ten focus groups of physicians stratified by self-identified race (black or white) and led by race-concordant moderators. Physicians were presented with a medical vignette about a patient (whose race was unknown) with Type 2 diabetes and untreated hypertension, who was also a current smoker. Participants were first asked to discuss what medical information they would need to treat the patient. Then physicians were asked to explicitly discuss the importance of race to the hypothetical patient's treatment. To identify common themes, codes, key words and physician demographics were compiled into a comprehensive table that allowed for examination of similarities and differences by physician race. Common themes were identified using the software package NVivo (QSR International, v7).

**Results:**

Forty self-identified black and 50 self-identified white physicians participated in the study. All physicians - regardless of their own race - believed that medical history, family history, and weight were important for making treatment decisions for the patient. However, black and white physicians reported differences in their views about the relevance of race. Several black physicians indicated that patient race is a central factor for choosing treatment options such as aggressive therapies, patient medication and understanding disease risk. Moreover, many black physicians considered patient race important to understand the patient's views, such as alternative medicine preferences and cultural beliefs about illness. However, few white physicians explicitly indicated that the patient's race was important over-and-above medical history. Instead, white physicians reported that the patient should be treated aggressively regardless of race.

**Conclusions:**

This investigation adds to our understanding about how physicians in the United States consider race when treating patients, and sheds light on issues physicians face when deciding the importance of race in medical decision-making.

## Background

The role of patient race and ethnicity in treatment decision-making has met considerable debate [1,2]. On one hand, clinicians sometimes use patients' race to predict disease risk and to determine appropriate drug therapy [3-5]. On the other hand, consideration of patients' race in clinical settings is also associated with bias and stereotyping, which can contribute to racial and ethnic healthcare disparities [6-9]. What we know about healthcare bias and race is further complicated by minority patients' perceptions of frequent discrimination. In both the United States and Europe, racial and ethnic minority patients report more discrimination and poorer quality of clinical care compared to non-minority whites [10-12].

Given the complexities of understanding medical and social contexts associated with race and racial bias in healthcare, it is critical to understand physicians' perspectives about when it is and is not appropriate to consider race when making clinical decisions for patients. Physicians' perspectives about race are particularly important to understand as the tangled web linking race and health continues to evolve, and as scientists continue to develop models of how race can be used to predict disease risk. What, then, do physicians think is the importance of race in treatment decision-making? Moreover, how do physicians consciously utilize race to make treatment decisions?

The purpose of this study is to explore United States primary care physicians' views about the importance of race in treatment decision-making by explicitly asking physicians if, and how race would factor into their decisions about treatment for a hypothetical patient. Using physicians' responses, we examined the importance of race, social, and medical factors in treatment decision-making, and explored differences in beliefs between black and white physicians.

## Methods

### Study Design

This investigation was part of the Physicians' Understanding of Human Genetic Variation (PUHGV) Study at the Social and Behavioral Research Branch of the National Human Genome Research Institute, National Institutes of Health (NIH). Aims of the PUHGV project were to acquire information regarding physicians' knowledge of human genetic variation [13], their beliefs about the relationships between genetics, race and medicine [14], and their use of race in clinical practice. In the present study, we consider the last PUHGV project aim - physicians' attitudes regarding the relevance of patients' race and other social attributes (beliefs, cultural practices, and socioeconomic status) in treatment decision-making.

### Sampling and Recruitment

A list of eligible physicians was generated using the American Medical Association Physician Profile Database of general internists, as well as identification of practicing physicians at Departments of Internal Medicine for the medical schools in each of the five study locations in the United States (Atlanta, GA; Detroit, MI; Los Angeles, CA; Philadelphia, PA; and Baltimore, MD). Using this list, general internists were recruited through invitation letters sent by mail or email. Because of underrepresentation of black general internists in the United States, we also notified local chapters of the National Medical Association (the largest and oldest national organization representing African-American physicians and their patients in the United States) in each of the five metropolitan areas of the study, and requested names of potentially eligible physicians; Snowball sampling was used to recruit additional black physicians, until project staff was able to gain a minimum of 12 physicians per focus group. The goal was to recruit 12 physicians per focus group in order to seat at least 8 to 10 participants, expecting 2 - 4 "no shows". Additional information regarding the PUHGV study, recruitment, and data collection are available in previously published literature [13,14].

### Human Participant Protection

Informed consent was obtained from each focus group participant before the start of the study, and a monetary incentive of $250.00 was provided. The study was approved by the institutional review board of the National Human Genome Research Institute of the National Institutes of Health.

### Data Collection

Ten focus groups were conducted with self-identified black or African American and self-identified white or Caucasian, board-eligible or board-certified general internists between October 2005 and March 2006. The focus groups were stratified by physician race. Two general internists who were experienced in focus group research methods served as racially concordant moderators for the focus groups; the same moderators were used in each of the five study locations.

### Vignette

To elicit discussion about physicians' attitudes about the use of race in treatment decision-making, physicians were presented with a brief clinical vignette describing a hypothetical patient. The patient's race was not provided to the physicians as part of the vignette. The clinical vignette read:

"Michelle is a 57-year-old patient who is new to your practice. Upon review of her medical records from an outside physician, you note that she has a history of untreated hypertension. Per the medical records, her blood pressure ranged 145 over 90 and 175 over 105 on two occasions. She has a history of type 2 diabetes; she smokes cigarettes, about a pack per day. Her current HDL is 35 and her LDL is 162. Michelle has health insurance through her employer, General Motors."

### Probes

First, physicians were asked to discuss what information they would want to have to make decisions about treating the patient. Then, the moderator of each focus group explicitly asked the physicians about their opinions on the relevance of race to the patient's treatment. Some questions included: What further information would you like to have to treat Michelle? How might race and ethnicity play a role in treatment of Michelle? How is race used in your clinical practice? When do you think race is medically relevant? Using the responses to these questions, we examined physicians' attitudes about the use of race in clinical practice, as well as of patient social attributes that physicians used to inform their decision-making.

### Analysis

Audio tapes of all focus group sessions were transcribed verbatim. Notes taken by trained observers at the focus groups sessions were also used to ensure accuracy of the transcripts. Transcription and coding for the initial PUHGV study is fully described in a previous publication [13].

For this analysis, transcripts were coded and reviewed by the vignette study core research team (authors S.A.S, A.O.O., J.C.F, and V.L.B). Several steps were taken to ensure careful, systematic qualitative analysis. First, two team members independently coded the vignette portion of each focus group transcript line by line using "open coding" [15-17]. "Open Coding" consisted of using salient key words and phrases that emerged from the focus group transcripts to formally identify categories and concepts relevant to the primary research questions: What do physicians think is the importance of race in treatment decision-making? How do physicians consciously utilize race to make treatment decisions?

From the open coding, a comprehensive set of codes was created. The core vignette study research team then developed a condensed list of the most significant codes relevant to the research question. The condensed list of codes was agreed upon by the entire research team. Coding differences were reconciled over the course of several meetings of team members, held to handle discrepancies in coding and to revise the coding scheme as needed. Finally, the vignette core research team compared codes and responses until consensus was reached for all transcripts.

NVivo (QSR International, v7) was used to code and qualitatively analyze the vignette transcripts. Using NVivo, the vignette core research team ran coding reports by physician race to create two data sets - one for black physicians and the other for white physicians, which were then used to compile comprehensive tables that highlighted the number of coding references (words, phrases, or statements) associated with each code. Repeated codes of the same participant and within the same broad category were treated as one instance. Next, data were analyzed by physician age, gender, number of years in practice, and practice setting (academic vs. non-academic) for each race-based data set. Coding reports were then created based on tables of all queries in the race-based data sets and then for the complete dataset with all physicians. Analysis was an iterative process, reviewing coding reports of the queried texts, reading related transcript paragraphs, and re-reading all transcripts. Our analysis also involved paying careful attention to negative cases (e.g., race not important), which were addressed by examination and re-examination of every case discussion around the medical relevance of race. Negative cases were resolved by the vignette core research team through comparison of all code differences to see whether the emergent themes were applicable to the majority of cases. Once negative cases were noted, we re-examined our codebook adding codes for negative cases. For example, codes were created for discussion around when race is not considered medically relevant. Re-coding of negative cases was conducted by the analysis team until properly coded. This iterative process continued until it was determined that there were no negative or unresolved cases.

Differences in discussions by physician race were determined by number of coding references, content, density, and breadth of discussion. Responses with the most coding references and most extensive discussion were listed as major themes.

## Results

### Demographic Characteristics

Ninety physicians participated in this investigation. Fifty of the participants self-identified their race as white, and 40 self-identified their race as black. Seventy-three percent (n = 66) of the physicians were male. The mean age of all physicians was 48 years. Forty-nine percent of all physicians practiced in an academic clinic (55% and 45% of black and white physicians, respectively). Other demographic characteristics of the participants are summarized in Table 1.

**Table 1:** Demographic Characteristics of Physicians

	% of Physicians		
	
Characteristic	Black (n = 40)	White (n = 50)	Total (n = 90)
Gender			
Female	40%	16%	27%
Male	60%	84%	73%
Age, years			
29-40	23%	22%	22%
41-50	44%	32%	37%
51-79	33%	46%	40%
Median years (yrs) in practice	14 yrs	17 yrs	15 yrs
Academic practice setting	55%	45%	49%
Distribution of patients			
> 50% white	10%	64%	40%
> 50% black	73%	20%	43%
> 50% Latino or other	18%	16%	17%

### Focus Group Findings

We found three main themes. Overall, we found that both black and white physicians held similar views that the patient's medical information (e.g. past medical history, family history) is the most important factor for treatment decision-making. However, black and white physicians' views about the medical relevance of race differ, and are presented as separate themes. Among white physicians, patient race was viewed as relatively unimportant for treatment decisions. Instead, white physicians believed that the hypothetical patient should be treated aggressively regardless of her race. Conversely, black physicians in the study believed that race is important for treatment decision-making, provides useful information for choosing medication, understanding disease risk, and is associated with social determinants (socioeconomic factors and cultural beliefs about illness) for the patients' health. These findings are described in detail below.

#### Theme 1. Black and White Physicians Hold Similar Beliefs that Medical Information is the Most Important Factor for Medical Decision-Making

Both black and white physicians reported that the medical and clinical history of the patient was important for treatment decision-making. Specifically, physicians considered it a priority to know the patient's medical information, family history, and weight and body mass index. When asked by the moderator, "What kind of information would you like to know to treat Michelle, the hypothetical patient?," physicians immediately discussed details of the patient's medical history, such as disease history, her past diagnostic and treatment history, and her current or past medication regimen. For example, two physicians noted:

"...it would be nice to know what prescriptions she's on currently. And, if she's been on other medications previously and what's happened over the years." (Black physician, Detroit Focus Group)

"I'd want to know what's been done already and what hasn't been done, who is she seeing, what medicines she's tried, whether it's a compliance issue, whether it's a tolerance of medicine issue, whether she hasn't had healthcare." (White physician, Baltimore Focus Group)

Physicians also discussed wanting to complete further diagnostic testing, such as urinalysis, chest radiography, and electrocardiography. Physicians explained,

"My feeling is [that] I would need to see her and probably do a complete work up on her... from the basic history and everything... as well as doing EKG[electrocardiogram], doing blood work, additional blood work, other than what's there, to determine where her base line is." (Black physician, Philadelphia Focus Group)

"...Physical exam findings to suggest longstanding diabetes or hypertensive changes." (White physician, Atlanta Focus Group)

Family history of disease was also important to physicians. The majority of participants indicated that having knowledge of the patient's family history could help assess the aggressiveness of treatment. Representative comments include:

"Family history... In terms of the risk factors that you described -- hypertension, diabetes, lipidemia. Even more than that is the end results of these -- like heart attacks and strokes and at what ages the family may have had it. Is it first degree or distant, remote relatives?" (White physician, Los Angeles Focus Group)

"Well it kind of gives you an idea of severity, of how urgently [you] need to get at her problem because of the family history. ...where persons die early in her family from [the disease], then you have to think about be[ing] more aggressive in managing her diabetes and hypertension." (Black physician, Baltimore Focus Group)

Finally, physicians indicated that weight and body mass index were critical and had an impact on the patient's treatment.

"Did you [referring to the moderator who read the vignette] say what she weighs?" (White physician, Detroit Focus Group)

"I'd also like to know her weight."(Black physician, Philadelphia Focus Group)

Another agreed, saying, *"Exactly, I think it [her weight] has an impact on all of her conditions." (Black physician, Philadelphia Focus Group)*

#### Theme 2. White Physicians Reported That Patient Race is Not Important for Treatment Decision-Making, and That Medical History Should Drive Decision-Making

Physicians were asked to be explicit about the ways in which race might be medically relevant in their clinical practice to deliver appropriate care to their patients, using the example of the hypothetical patient vignette. The majority of black physicians in each focus group stated that knowing the hypothetical patient's race would affect treatment decisions. Among white physicians, however, few indicated that the patient's race was important for treatment decision-making.

There were marked differences regarding the importance of race between black and white physicians. Of the five focus groups among black physicians, all had extensive discussion regarding the importance of race for medical decision-making. However, only four white physicians discussed the importance of the patient's race for medical decision-making. Responses of white physicians who supported the use of race for treatment decision-making are below.

*"Let's take African-Americans. I've always stressed to them that because they're African-American, for instance, treating hypertension, that they really have to pay attention and take their medications and let them be on the team with me. It's because they're African-American, we make them realize it that they really have to pay attention. I think it's a good thing that we go into their race." (White physician, Los Angeles Focus Group*)

"I would want to know the race -- I'm not doing hospital work anymore, but if someone was presenting me a case, I'd want to know the race so you would have an idea of how they grew up. I mean what their diet was, what their socioeconomic and the milieu was as they were growing up." (White Physician, Philadelphia Focus Group)

During conversations regarding the importance of race for treatment decision-making, white physicians sometimes indicated that race and ethnicity were important for treatment of disease, but immediately followed by stating that race was not as important as other factors. For example, two physicians said,

"If she was Hispanic, diabetes seems to be more malignant in terms of its course. African-American hypertension seems to be a more difficult disease to treat, and obesity seems to be more prevalent. But in terms of overall, I think basic nutrition is really one of the key problems we see in our total culture. It's not raced based or ethnic based. It's pretty much the commercial world we live in." (White physician, Los Angeles Focus Group)

"My initial thought is that it [race] doesn't affect it [her treatment] very much, at least, initially. That the initial evaluation and the initial treatment is going to be pretty much the same. And race is a secondary or tertiary or farther on down the list than the other information that I need to have at hand in order to make the initial treatment decisions. Yes, it may be a factor. It may come into play more later on but not in the beginning on the first visit." (White physician, Atlanta Focus Group)

In the Los Angeles focus group only, white physicians warned one another regarding the use of race in clinical settings.

"But you still have to be careful. I mean if you tell somebody too many negatives about their race or something like that, they might take it the wrong way. That's what he's referring to." (White physician, Los Angeles Focus Group)

"I have some apprehension because there is a perception among some patients that you ask these probing questions, they might be sensitive to it. I mean I have this fear sometimes that I'm going to offend somebody by singling them out, their group." White physician, Los Angeles Focus Group)

Overall, the majority of white physicians stated that while race is sometimes important for understanding disease-risk, diet and socioeconomic status, race in the clinical vignette provided little information over medical history. Given the severity of Michelle's condition, knowing her race would not provide any additional helpful information, and most white physicians agreed that she should be treated aggressively regardless of her race. Some white physicians stated,

"I'm not sure that it's [race is] relevant in this woman's case...because of all the medical issues that you've described, she's going to need to be treated aggressively." (White physician, Philadelphia Focus Group)

"I'm more concerned with was she compliant in the past? Was she on medications at all and wasn't taking them for some reason rather than how her race factored into this?" (White physician, Atlanta Focus Group)

"I agree. I think your endpoints don't change and too, I think, just knowing their race, you have to avoid making generalizations about the race. Every individual is completely different. So, I kind of agree that it is not as important, at least to me." (White physician, Los Angeles Focus Group)

#### Theme 3. Black Physicians Reported That Patient Race is Medically Relevant and Can Be Useful in Treatment Decisions

Many black physicians explicitly indicated that patient race would be important. There was general consensus among black physicians that race is an important indicator that should be used in treatment decision-making. Some black physicians stated,

"*I think being an African American is a risk factor in and of itself. And, I think that when you see an African American then you need to often be more aggressive than you would and use different standards than you would for the general white population." (Black physician, Philadelphia Focus Group)*

*"I think it's very significant to know what her race is because it will make some decisions... about what paths I'm going to use to treat her..." *and *"I mean [race] is important to choosing the medication." (Black physician, Atlanta Focus Group)*

"[It is] important to choosing the medication, race and ethnicity... I think it's also important as [to] how you approach her." (Black physician, Detroit Focus Group)

Black physicians also stated that treating black patients more aggressively is necessary because of co-morbidities or increased risk for developing secondary conditions, saying,

"In African Americans, hypertension... [and] diabetes [are] more significant than that as compared to who is white or of a different race [because of] more severe problems with kidney control, kidney function." (Black physician, Baltimore Focus Group)

Black physicians also noted that patient race guides their ability to become aware of the patient's socio-cultural context. Specifically, black physicians discussed the importance of the patient's beliefs, health practices, and repeatedly inquired about the patient's socioeconomic position. Physicians were interested in the patient's ability to afford consistent care, maintain health insurance, and pay for prescription medications. Black physicians also noted that being aware of socio-cultural beliefs and practices that may affect health care would facilitate patient-physician communication and ultimately treatment decisions. One black physician stated,

"Cultural issues... are relevant no matter who you are. You know, [there is] no telling what this patient [is using to treat her illness]. I was thinking maybe they are treating their blood pressure with medicine or with some herbs..." (Black physician, Detroit Focus Group)

Black physicians also reported wanting to know about the patient's beliefs about illness, views on taking medication, experiences with disease and priorities with respect to disease management. As one black physician said,

"I'd be curious what she thinks about these issues [her health]. Does she think they're important? Does she think they're real? ...I need to know what she's thinking." (Black physician, Philadelphia Focus Group)

Physicians who deemed social attributes important in clinical decision-making discussed that knowing about the patient's beliefs could provide understanding into how the patient conceptualized her disease.

"[I want to know] her opinion on if she thinks this is important. And, it may be something low on her priority list... as far as being treated for hypertension or diabetes. She may not think it's important. That may be why she hasn't followed up. I think you need to explore that aspect of it as well."(Black physician, Detroit Focus Group)

In contrast, very few white physicians mentioned the importance of the patient's social characteristics. Out of all of the white physician focus groups, there were only four quotes regarding social determinants that might influence treatment decision-making. These four quotes are:

"You need to treat the person aggressively, but they may not be open to it. They may have a different view of the world." (White physician, Los Angeles Focus Group)

"I want to know her social history -- who is she living with. Is she the primary breadwinner? What are her psychosocial stressors." (White physician, Los Angeles Focus Group)

After being probed by the focus group moderator, two physicians stated,

"I'm thinking on the average, African-Americans are less likely to have insurance, less likely to have as many assets for paying for medications. And I've never really felt I'm doing somebody a great job if I come up with my brilliant idea on what they should do when I give them a prescription they can never pay for. So, you want to try to take that into account if your goal is to actually accomplish something rather than just give somebody a piece of paper." (White physician, Atlanta Focus Group)

"In your case report we started the conversation with, I think we all agreed that that patient was a walking time bomb. For me the interesting question may or may not be related to race -- it's why did she walk into the office at that point and still have no treatment. So, one of my goals is always to try to understand what motivates people -- you know, their paradigm of their own health. And that may or may not be a race-related issue. " (White physician, Los Angeles Focus Group)

Further analysis examined themes across other physician attributes, including age, sex, percentage of minority patients, number of years in clinical practice, practice setting and region where physicians practiced. We found no noteworthy differences in any focus group themes by these physician characteristics except one, a difference based upon practice setting for black physicians (academic vs. non-academic). Our analysis revealed that black physicians' practice setting - academic or non-academic - influenced their views about the importance of the patient's social attributes. While all black physician focus groups included discussion regarding the patients' social attributes, the majority of the discussion about affordability of medication and healthcare was among black physicians that practice in academic settings.

## Discussion

Our study explored beliefs that black and white physicians have about the importance of patient race for treatment decision-making. Although patient medical history was important to both groups of physicians, we found noteworthy differences in how black and white physicians viewed the importance of patient race. Specifically, we discovered that black physicians in our sample viewed race as important for treatment decision-making, while only four white physicians considered race medically relevant.

Our data suggest that black physicians view race as important when making decisions about their patients' treatment. In contrast to most white physicians, many black physicians viewed race as important for choosing medications and assessing risk of disease. It should be noted, however, that both groups - black and white physicians alike - viewed medical history as most important for medical decision-making. This finding has important implications, since probability-based models suggest that race should be a primary proxy for establishing that any given patient of a particular racial or ethnic group will experience a health problem. For example, since African Americans in the United States suffer from increased risk of hypertension and diabetes [18], disorders outlined in our clinical vignette, some reasoning suggests that population-based probability of disease should accompany the decision-making process. However, we found that white physicians in our study did not rely on race as a determinant for treatment decision-making. Other studies agree with this, finding that among a largely white physician population, doctors rarely mentioned race and ethnicity to determine clinical assessments [19]. Moreover, while black physicians indicated using race as a proxy for disease risk, black physicians held nuanced and complex views about the appropriate context in which race should be used (e.g. to determine appropriate medication and to understand social determinants of health linked with stress and health disparities).

Black physicians also linked race to the hypothetical patient's social determinants of health such as socioeconomic status, as well as their own ability to deliver culturally appropriate care (i.e., being knowledgeable, as well as responsive to the patient's cultural beliefs about illness). Other studies are consistent with our findings. For example, a national survey of doctors suggests that black physicians have greater knowledge and awareness of health and healthcare disparities that affect minority populations [20]. Our findings might be further explained by evidence that minority physicians provide care in underserved areas for minority patients at higher rates than non-minority physicians. In our study, 36% of white physicians served a minority patient population, while 73% of black physicians served a minority patient population. This difference may have influenced the discussion about race and social determinants of health among physicians in the study, who may have filled in details about the clinical vignette based on their own experiences.

In depth analysis of black physicians' discussion of social determinants of health revealed differences based on physician-level attributes. When compared to black physicians who did not work in an academic setting, those whose medical practice setting was linked to an academic institution more frequently expressed views about the importance of the patient's social determinants of health. Black academic physician participants articulated that race was associated with cultural values related to health, and especially emphasized access to consistent, affordable care.

We also offer possible explanations regarding the fewer occurrences of discussion regarding the use of patient race in treatment decisions among white physicians in our study. Although multiple responses from the same individual were only counted once, it is possible that the number of mentions of particular codes regarding the use of race reflect how talkative physicians were in some focus groups versus others. However, when we assessed differences in the coding across all focus groups, there were marked thematic differences between black and white physicians. All five focus groups among black physicians contained discussion about the importance of race for medical decision-making, regardless of length of discussion. Conversely, all focus groups among white physicians indicated that race is not especially medically relevant.

The differences in black and white physicians' responses about race for treatment decision-making may also indicate that, compared to black physicians, white physicians were less comfortable discussing issues related to race. Other evidence supports this as a possible factor. For example, Littleford et al (2005) found that whites experience greater discomfort than minorities when discussing issues of race and ethnicity in a group setting [21]. In our study, white physicians voiced concerns about the potential negative effects of incorporating race into decision-making for their patients. White physicians in our study specifically cautioned one another to be careful about stereotyping patients based on race, or about minority patients being offended by the topic of race being raised.

Although white physicians may have felt less comfortable discussing race compared to black physicians, our focus group protocol was organized to encourage free-flowing discussion of race. The study design was careful to include same race participants and race-concordant physician moderators since choosing moderators with similar characteristics to focus group participants is an effective strategy toward reducing discomfort when discussing sensitive issues [22,23]. In addition, the physician moderators followed identical protocols and guides in both black and white focus groups, which included the same questions and probes. Nonetheless, some white physicians may have been cautious about discussing issues of race.

The differences we observed in discussions held by black and white physicians about the importance of race in treatment decision-making may also reflect that, in some cases, white physicians may not consciously view race as an important factor for treatment decision-making. Still, some evidence indicates that while white physicians may consciously state that race is not of importance for treatment decision-making they subconsciously use race to make decisions. For example, a study by Green et al. [7] indicates that while most white physicians do not admit having different feelings toward and perceptions of blacks and whites explicitly, their implicit measures show some degree of unconscious use of race in the form of bias in treatment decisions, favoring whites over blacks. Our study did not measure racial bias among physicians, nor do our results suggest that white physicians in our sample display racial bias in delivery of care.

We note, finally, that perceptions of black physicians regarding the importance of patient race may have also been influenced by their own experiences as members of a racial minority group [24]. Evidence suggests that black physicians report seeing, or experiencing discrimination ascribed to their racial assignment, while whites think of overt expressions of racism as rare occurrences in our society [25,26]. Nunez-Smith et al. (2008) reported that black physicians sometimes find it their place to protect their minority patients from discrimination in healthcare settings [27]. Therefore, it is not surprising that black physicians in our study view race as an important determinant of health to be considered in treatment decisions. Consistent with other research [23-26], our findings suggests that the role of physician race in reducing health disparities cannot be ignored, and that racial and ethnic U.S. trained minority physicians may play a particularly important role for understanding racial and ethnic minority patients' social experiences in the United States.

### Strengths and Limitations

This study is one of few to probe how and why physicians might consider race when treating patients with complex medical problems (i.e., chronic disease, high risk health behavior such as smoking, etc.). Still, some limitations of this work should be considered. First, participants were not randomly selected and were limited to black and white physicians from particular geographic regions in the United States. Second, focus group studies are generally characterized by small sample sizes and may not be representative of the population. Moreover, our results are exploratory in nature and quantitative methods were not used to compare differences between groups. These design and sampling strategies may reduce the external validity of our study.

Also, our exploration of clinicians' intended use of race used a clinical vignette with self-report of intent rather than observation of actual clinician behavior. It is important to note that our clinical vignette focused on a hypothetical patient with Type 2 diabetes and untreated hypertension, who was also a current smoker. Thus, physicians' responses about the relevance of race for this patient's treatment may not represent physicians' responses for other types of clinical cases or illnesses. Finally, while we were able to elicit physicians' attitudes regarding the use of race in clinical settings, perceptions about race influence decisions both consciously and sub-consciously, and we did not measure subconscious attitudes about race. Our findings must be interpreted in light of these limitations.

## Conclusions

Our study used an in-depth, qualitative approach to learn from physicians' own responses about how race is sometimes used in treatment decision-making. This approach revealed new information not only about patient race and its use for treatment decisions, but also prompted valuable discussions regarding the importance of patient social attributes that physicians use to inform their clinical decision-making. This study's unique contributions to the literature on health disparities and inequities are: (1) an in-depth, qualitative analysis of the value that white and black physicians in the United States place on race and other patient social attributes for medical decision-making, and (2) the discovery that physicians' race and practice setting (academic versus non-academic) are potentially relevant to their views about what information is important in making culturally-appropriate patient treatment decisions. This study represents an important first step that we hope will lead to better understanding of how race is perceived, and how race is used by physicians for treatment decisions. We recommend that future work explore questions on treatment differences, race, and health though (1) use of quantitative and qualitative methods to better understand how race and other patients' social attributes influence physicians' decision-making and their delivery of culturally-sensitive care; (2) exploring physicians' thought process regarding race and disease risk, and drug therapy; and (3) building evidence on how physicians think about, and link race and genetic variation in clinical settings.

## Competing interests

The authors declare that they have no competing interests.

## Authors' contributions

SAS led the writing team in the manuscript preparation, as well as contributed to all aspects of the qualitative analysis including coding, grouping codes by attribute, and developing themes from the data. SLS contributed significantly to paper development and study design. AOO participated in study design and preliminary analysis and paper for content. LAC contributed significantly to paper development and revised critical sections of the paper for content. JCF contributed to all aspects of preliminary qualitative analysis and early drafts of the paper. VLB conceived of the study, participated in design and coordination of the analysis, and assisted in all drafts of the manuscript. All authors approved the final manuscript.

## Funding

This research was supported in part by the Division of Intramural Research of the National Human Genome Research Institute, National Institutes of Health. Writing support was granted in part by an EXPORT Center of Excellence grant provided by the National Center on Minority Health and Health Disparities, National Institutes of Health (5-P60-MD000503), and the W.K Kellogg Foundation (Grant #: P0117943). Revisions of the manuscript, edits and preparation for submission were funded by the Post-doctoral Fellowship, University of Texas School of Public Health Cancer Education and Career Development Program - National Cancer Institute/NIH Grant R25-CA-57712. The content is solely the responsibility of the authors and does not represent the official views of the National Human Genome Research Institute, National Cancer Institute, National Institutes of Health, or Department of Health and Human Services.

## Pre-publication history

The pre-publication history for this paper can be accessed here:

http://www.biomedcentral.com/1472-6963/11/183/prepub
